# Pathogenicity and virulence of Kyasanur Forest disease: A comprehensive review of an expanding zoonotic threat in southwestern India

**DOI:** 10.1080/21505594.2025.2580154

**Published:** 2025-10-30

**Authors:** Himanshu Kaushal, Virendra Kumar Meena, Shalini Das, Sritama Sarkar, Ramesh S Kartaskar, Vikas Sharma, Naveen Kumar

**Affiliations:** a ICMR-National Institute of Virology, Pune, India; b Academy of Scientific and Innovative Research (AcSIR), Ghaziabad, India; c Taluka Health Office, Sindhudurg, India

**Keywords:** Kyasanur Forest disease, vaccines, genetic diversity, host immune responses, clinical features

## Abstract

Kyasanur Forest Disease (KFD) is a tick-borne viral hemorrhagic fever caused by the KFD virus (KFDV). First identified in 1957 in Karnataka, India, KFD primarily affects humans and non-human primates. The disease has a broad clinical manifestation, ranging from asymptomatic infections to severe hemorrhagic complications, with a mortality rate of 3–10%. Over time, KFD has shown a gradual geographical expansion, with cases now reported beyond Karnataka. KFD has been a public health challenge for nearly seven decades, as no effective vaccine or antivirals are available. Although humoral immunity is critical in controlling KFD infection, with neutralizing antibodies conferring durable protection, the role of cellular immunity is not well studied. Preventive strategies – such as tick control, community education, and well-planned surveillance – are vital to reducing disease burden. This review synthesizes knowledge on KFD, encompassing its discovery, epidemiology, clinical presentations, diagnostic assays, viral evolution, pathophysiology, immune mechanisms, interventions, and research priorities.

## Introduction

Kyasanur Forest Disease (KFD) is an acute viral hemorrhagic fever affecting humans and monkeys. The etiological agent, KFD virus (KFDV), is a tick-borne flavivirus first identified in the Western Ghats of southern India [1]. The disease is primarily transmitted by the hard tick *Haemaphysalis spinigera* [2]. KFD manifests a spectrum of clinical presentations in humans, ranging from asymptomatic infections to mild illness and severe hemorrhagic complications that can prove fatal [3,4]. While the majority of patients recover fully, approximately 10–20% develop a more severe biphasic illness. The overall case fatality rate for KFD ranges from 3–10% [5–9]. The geographic distribution of KFDV, limited initially to Karnataka state in India, has expanded to encompass neighboring states including Tamil Nadu, Kerala, Goa, and Maharashtra [10–14]. Despite being recognized for nearly seven decades, KFD is still a public health concern in southern India, with no effective interventions. Recent studies have further highlighted the inadequate effectiveness of the licensed vaccine in preventing KFD infection [9,15]. This comprehensive review explores critical dimensions of KFD, including its discovery as a novel pathogen, clinical features, viral evolution, diagnostic methods, pathophysiology, host immune dynamics, virus evolution, vaccination approaches, antivirals, prevention strategies, and future research priorities.

## Early investigations

On 23 March 1957, health officials of Karnataka state in India notified the Virus Research Centre (VRC), now the Indian Council of Medical Research (ICMR)-National Institute of Virology (NIV), Pune, about mysterious monkey deaths occurring in the Kyasanur forest region of Shimoga District, Karnataka state (then Mysore state), India [1]. On March 26, the District Health Officer reported that several villages near these forests were experiencing cases of an extended febrile illness, initially suspected to be enteric fever. Local villagers had begun referring to this disease as “the monkey disease.” Initially, investigators hypothesized that humans and monkeys might have contracted an illness from contaminated water sources. This idea originated from the observed behavior of wild monkeys in India, which often share water sources with humans, especially during the summer when both rely on the same tanks and rivers for drinking water. In response to this presumed epidemic of enteric infection, public health authorities implemented multiple control measures. These included mass typhoid TAB vaccine administration, well water chlorination, dieldrin spraying for fly control in affected villages, and treating symptomatic individuals with chloramphenicol [1].

Later, investigators considered yellow fever a potential cause of the outbreak, as it was the only known infection that could cause widespread mortality in humans and forest monkeys with similar epidemiological patterns [16]. This idea was particularly concerning since yellow fever had never been documented in the Indian subcontinent. However, this hypothesis was rejected as multiple attempts to isolate the Yellow fever virus (YFV) from mosquito pools collected in affected areas proved unsuccessful [1].

A breakthrough came when researchers successfully isolated viruses from three dead monkeys from Kyasanur Forest and two human patients suffering from severe febrile illness [1,17,18]. Subsequent hemagglutination inhibition tests revealed that the villagers’ illness was caused by a Group B arthropod-borne virus [1,19]. Complement fixation tests using an antigen prepared from the virus, isolated from an infected monkey, confirmed that humans and monkeys were affected by the same infection [1]. Further analysis identified the virus as a member of the Far-Eastern tick-borne complex (previously Russian Spring-Summer tick-borne complex) [18,20]. Additionally, the observation of *Haemaphysalis* ticks on the monkeys caught from affected regions of the Kyasanur forest suggested that the viral agents might have originated from Siberian regions, potentially transported by migratory birds. To investigate this theory, researchers examined thousands of migratory birds for ticks. However, they found only one bird carrying a tick of the genus *Hyalomma*, a species present both within and outside India [7]. This limited evidence made the theory of infected tick transfer by migratory birds unlikely.

Entomological investigations conducted in Kyasanur Forest during April 1957, at the peak of human cases and monkey deaths, revealed significant findings about potential disease vectors. Researchers found that there were almost no mosquitoes biting during the day at ground level, and no separate group of mosquitoes lived in the forest canopy. Other biting *Diptera*, including *Phlebotomus*, *Simulium*, and C*ulicoides*, were not found attacking humans in the forest [20]. The slow linear spread of KFD, combined with these observations, suggested that the vector was likely another arthropod with limited mobility. The discovery of *Haemaphysalis* ticks on infected monkeys prompted an intensive tick collection effort in the affected forest areas. Researchers gathered approximately 20,000 ticks of various life stages within the first few months. During April, when human cases were particularly numerous, *Haemaphysalis* larvae and nymphs comprised nearly 90% of all ticks collected through forest drag sampling [2,20]. Subsequent research yielded repeated virus isolations from *Haemaphysalis* ticks collected in areas with human and monkey infections, while ticks from adjacent unaffected areas tested negative [20]. This pattern strongly indicated *Haemaphysalis* ticks’ role in virus transmission. Based on these findings, researchers named the pathogen “Kyasanur Forest Disease virus (KFDV),” and the resulting human illness became known as “Kyasanur Forest Disease (KFD),” both named after the location where the virus was first identified.

## Epidemiology and geographical expansion of KFD

**Figure 1.**
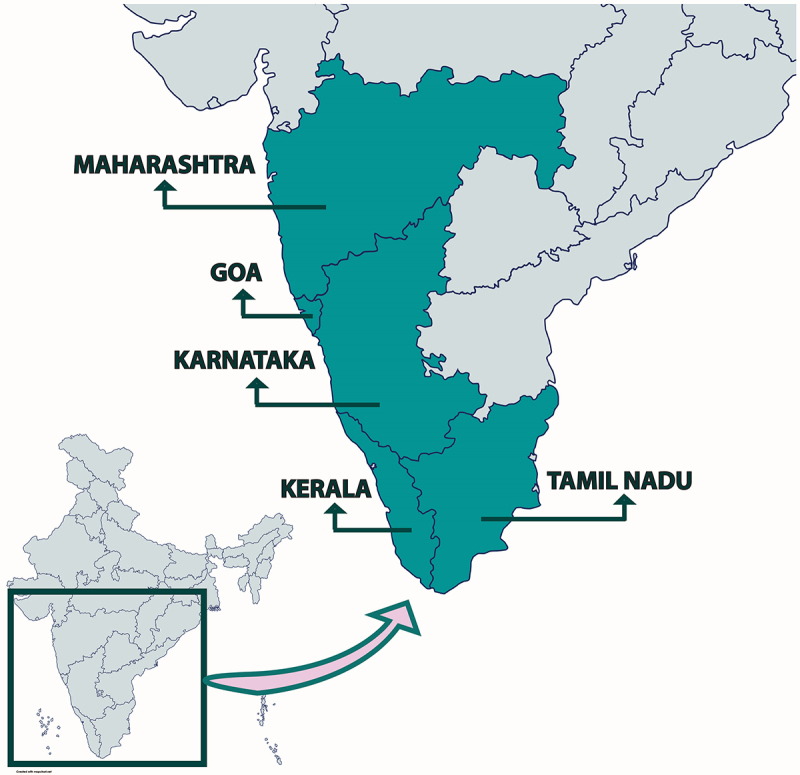
Indian states with confirmed cases of KFD. The map highlights Indian states with laboratory-confirmed cases of KFD, indicating the geographical spread and emergence of the disease beyond its traditionally endemic regions.

KFD was identified in 1957 in the Shimoga district of Karnataka, India, where it remained localized for over a decade [7]. Subsequently, the disease spread to neighboring districts of Karnataka, which include Uttara Kannada, Dakshina Kannada, and Udupi, marking its initial geographical expansion phase [7]. Subsequent decades saw KFDV transcend state boundaries, with the virus detected in the autopsy samples of monkeys and tick pools in Tamil Nadu’s Nilgiris district in 2012 [13,21]. Human cases emerged in Kerala in 2014 and later in Goa and Maharashtra during 2015–2016, resulting in 17 fatalities collectively [10,12,22,23]. As of 2024, KFD remains endemic to five Indian states – Karnataka, Tamil Nadu, Kerala, Goa, and Maharashtra – with recent expansions into Karnataka’s Hassan and Mysore districts [9] (Figure 1).

**Figure 2.**
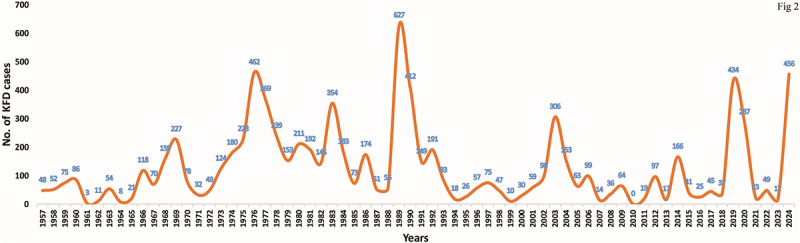
Temporal distribution of KFD cases in Karnataka, India. The figure depicts the distribution of reported KFD cases in Karnataka, India, from 1957 to 2024, highlighting fluctuations in disease incidence nearly seven decades.

Approximately 500 human KFD cases are reported annually, with a case fatality rate of 3–10% [5–9]. Surveillance data from India’s Integrated Disease Surveillance Programme (IDSP), National Health Mission, and National Centre for Disease Control (NCDC) highlight Karnataka as the most affected state, accounting for the majority of cases since 1957 (Figure 2) [21,24–26]. Although KFDV has so far been reported only in five Indian states, serological studies suggest that the virus may be silently spreading in other areas as well. Antibodies against the virus have been detected in Gujarat, Rajasthan, West Bengal, and the Andaman and Nicobar Islands, though cross-reactivity with other flaviviruses may partly explain these findings [21,27–30].

Ecological and anthropogenic factors drive KFD’s spread. Deforestation, encroachment into forested areas, and climate change are the key driving forces in disrupting tick-monkey transmission cycles and increasing human exposure [31–33]. Recent studies have identified landscapes with a mix of forest and plantations, high densities of indigenous cattle, and low coverage of dry deciduous forests as areas with elevated KFD risk [34]. Additionally, Hassall et al. (2023) emphasized the role of transovarial transmission and the involvement of small mammals and birds in maintaining KFDV, challenging earlier assumptions that primates are the primary drivers of infected tick distribution [35]. Despite existing efforts that focus on vector control and limiting human exposure, the effectiveness of current strategies remains uncertain due to limited knowledge of KFDV’s complex ecology involving vectors, hosts, and environmental factors [36]. Research priorities identified by Burthe et al. include clarifying the roles of cattle and monkeys in transmission and designing ecological interventions to block disease spillover [36]. The detection of KFDV in new geographic areas highlights the urgent need for strengthened surveillance, targeted vector control, and community awareness.

## Clinical spectrum of KFD

KFD exhibits a broad clinical spectrum, ranging from a mild, self-limiting febrile illness to severe hemorrhagic complications and, in some cases, death. While most infected individuals recover without significant sequelae [20,37], approximately 10–20% of symptomatic cases exhibit a biphasic illness, characterized by two distinct clinical phases [38]. In addition to symptomatic cases, asymptomatic and presymptomatic infections have been reported [1,4]. Isolation of KFDV from patients as early as 36–48 hours before the onset of symptoms supports the presymptomatic viremic phase, which has implications for transmission dynamics and surveillance [1]. Furthermore, detecting anti-KFDV IgM antibodies in apparently healthy individuals from endemic regions indicates the occurrence of asymptomatic infections, suggesting that the disease burden is underestimated [4]. Besides, some cases with KFD-like symptoms in endemic areas may remain unconfirmed due to the absence of laboratory testing, particularly in remote settings. This can lead to an underestimation of actual KFD burden and complicate the interpretation of clinical symptom patterns.

The first phase of illness is characterized by acute febrile illness, typically beginning 3–8 days following a tick bite and lasting 5–14 days [8]. The sudden onset of high-grade fever, severe myalgia, headache, and prostration marks this phase. Other accompanying features include gastrointestinal symptoms (nausea, vomiting, abdominal pain, diarrhea), conjunctival suffusion, palatal petechiae, and hepatomegaly [39,40]. Hemorrhagic manifestations (e.g. epistaxis, gum bleeding) are observed in about 10–15% of cases with KFD.

The second phase begins after 7–14 days of remission and occurs in approximately 10–20% of patients. Symptoms include recurrent fever, severe headache, neck stiffness, cerebellar ataxia, blurred vision, tremors, and, rarely, seizures [1,3,40,41]. This phase typically resolves within days to weeks, but may leave residual deficits like persistent ataxia in rare cases [8]. Risk factors associated with fatal outcomes include advanced age, myocarditis, altered sensorium, and abnormal laboratory findings [42]. Overall, KFD’s biphasic nature demands clinical vigilance. The acute phase emphasizes systemic and hemorrhagic complications, while the second phase focuses on neurological involvement.

Recent clinical data and practitioner reports suggest potential shifts in KFD presentation. A Kerala case series (2013–2023) reported a wider range of complications – including altered sensorium (82%), acute respiratory distress, myocarditis, hepatitis, and hemophagocytic lymphohistiocytosis – indicating increased clinical severity rather than new symptoms [43]. In contrast, the first documented outbreak in Goa showed an absence of hemorrhagic signs, suggesting possible regional and temporal variability [44]. While no systematic study has confirmed these trends, combined evidence from published work and clinician observations highlights the need for greater clinical vigilance and further research.

While KFD generally presents as a biphasic febrile illness, with most patients recovering within a few weeks, a subset experiences a protracted convalescent phase featuring persistent neurological symptoms such as hand tremors, unsteadiness, and prolonged lethargy that may endure for several weeks. However, true chronic sequelae are rare [8,45]. Although no formal DALY (Disability-Adjusted Life Year) estimates specific to KFD have been published, the prolonged convalescence and productivity loss suggest a substantial public health impact.

## Technological evolution in KFD diagnosis

The diagnosis of KFD has evolved from traditional methods to advanced molecular techniques, reflecting advancements in virology and diagnostics. Early methods relied on *in vivo* and serological assays, including inoculation of suckling mice with patient serum, hemagglutination inhibition (HI), complement fixation (CFT), and neutralization tests (NT) [1,5,19]. These techniques, while foundational, were time-intensive, required specialized facilities, and exhibited variable sensitivity. Peripheral blood samples from humans, non-human primates, and tick pools from endemic regions served as primary diagnostic materials [1,46].

Modern diagnostics prioritize reverse transcription-polymerase chain reaction (RT-PCR), which targets the KFDV NS5 gene. This method offers rapid, sensitive, and specific detection of viral RNA, outperforming traditional assays [28]. Therefore, it is the preferred assay for KFDV detection in clinical materials such as human blood or serum, blood and viscera from infected monkeys, and tick pools [28]. RT-PCR is the most effective assay during the acute phase of infection (first 4 days post-symptom onset), as viral loads peak in blood during this period [1,46,47]. However, its utility declines after day 10, as less than one-fifth of cases test positive beyond this window, though rare detections up to 18 days have been documented [47]. For later-stage diagnosis, an enzyme-linked immunosorbent assay (ELISA) is preferred to detect IgM antibodies against KFDV. IgM antibodies typically emerge 5–7 days post-onset, persisting for weeks [47,48]. This dual approach – RT-PCR for early infection and ELISA for later phases – ensures diagnostic accuracy across disease stages [48,49].

Recent innovations focus on point-of-care testing (POCT) to enhance field applicability. The Truenat KFD assay, a portable, microchip-based RT-PCR system, detects as few as 10 viral RNA copies and inactivates samples via buffer integration, improving biosafety [50]. Its battery-operated design, minimal infrastructure requirements, and rapid turnaround make it ideal for remote, resource-limited settings, underscoring its potential for outbreak response and surveillance [50]. The Truenat assay shows perfect concordance in testing human, monkey, and tick sample panels [50]; however, despite including tick pools in its validation, its performance on field-collected or low-viral-load tick samples remains insufficiently evaluated. Key barriers to broader deployment include cost and supply chain constraints, limited training of peripheral health workers, cold-chain requirements for reagents, and the clinical overlap of KFD with other febrile illnesses such as dengue and leptospirosis. In such contexts, practitioners often rely on syndromic diagnosis until laboratory confirmation is available, potentially delaying case detection and reporting.

## KFD virus: Taxonomy, structure, and genome

**Figure 3.**
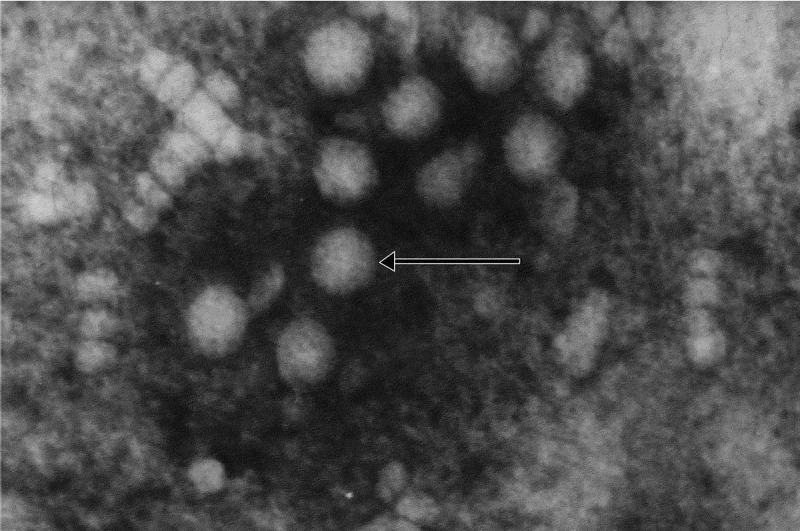
Transmission electron micrograph of KFDV. (Courtesy: Electron Microscopy & Histopathology Group, ICMR-NIV, Pune).

KFDV belongs to the family *Flaviviridae*, genus *Flavivirus*, and is a member of the Tick-Borne Encephalitis Virus (TBEV) serocomplex, which includes Alkhumra Hemorrhagic Fever Virus (AHFV) and Omsk hemorrhagic fever virus (OHFV) [8]. This classification reflects its close genetic and antigenic relationships with other tick-borne flaviviruses, sharing conserved structural motifs and evolutionary lineage. Structurally, KFDV is an enveloped, spherical virion measuring 40–65 nm in diameter [7,51]. The viral envelope, derived from host membranes, is studded with two glycoproteins: the envelope (E) protein and the smaller membrane (M) protein. The E protein mediates host-cell receptor binding, membrane fusion, and immune evasion, while the M protein facilitates virion assembly and maturation [51]. Beneath the envelope lies the nucleocapsid, which encapsulates the single-stranded, positive-sense RNA genome. This structural organization is conserved across flaviviruses, enabling efficient host-cell entry and replication. A representative transmission electron micrograph of KFDV prepared using negative staining is shown in Figure 3.

**Figure 4.**
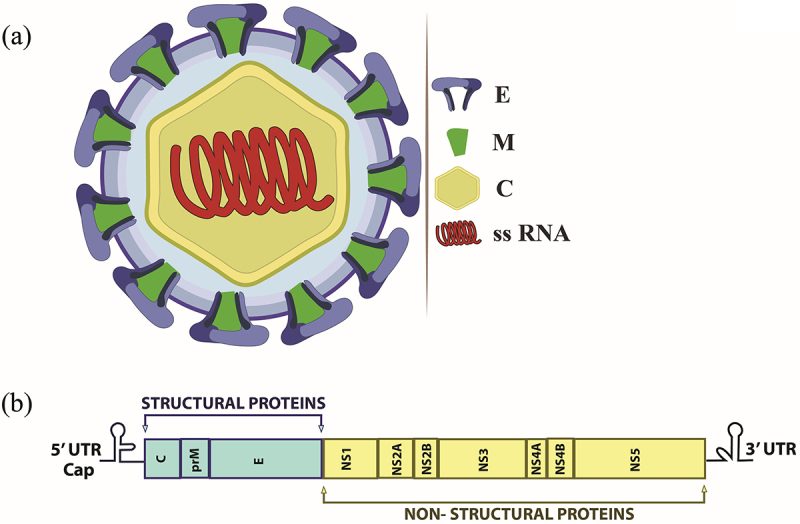
Structural overview of the KFD virus and its genome organization. (a) Schematic diagram of the KFD virion, showing the envelope (E), membrane (M), capsid proteins (C), and RNA genome. (b) Illustration of the KFDV genome, a single-stranded positive-sense RNA, highlighting its structural and non-structural protein-coding regions.

The KFDV genome comprises approximately 11 kilobases of linear RNA, featuring a single open reading frame flanked by 5’and 3’ untranslated regions (UTRs) [51]. The ORF encodes a polyprotein precursor that is post-translationally cleaved by viral and host proteases into three structural proteins (capsid [C], precursor membrane [prM/M], and envelope [E]) and seven non-structural (NS) proteins (NS1, NS2A, NS2B, NS3, NS4A, NS4B, and NS5). A schematic diagram of the KFD virion and its genome structure is depicted in Figure 4. The NS proteins orchestrate viral replication, immune modulation, and polyprotein processing, with NS3 functioning as a helicase/protease and NS5 as the RNA-dependent RNA polymerase. The UTRs form secondary structures critical for genome cyclization, replication, and interaction with host machinery [51]. Genetic studies highlight conserved regions in the E protein, a primary target for neutralizing antibodies, and variability in NS proteins, influencing viral adaptation and pathogenicity.

## Phylogenetic and sequence similarity insights into KFD virus

**Figure 5.**
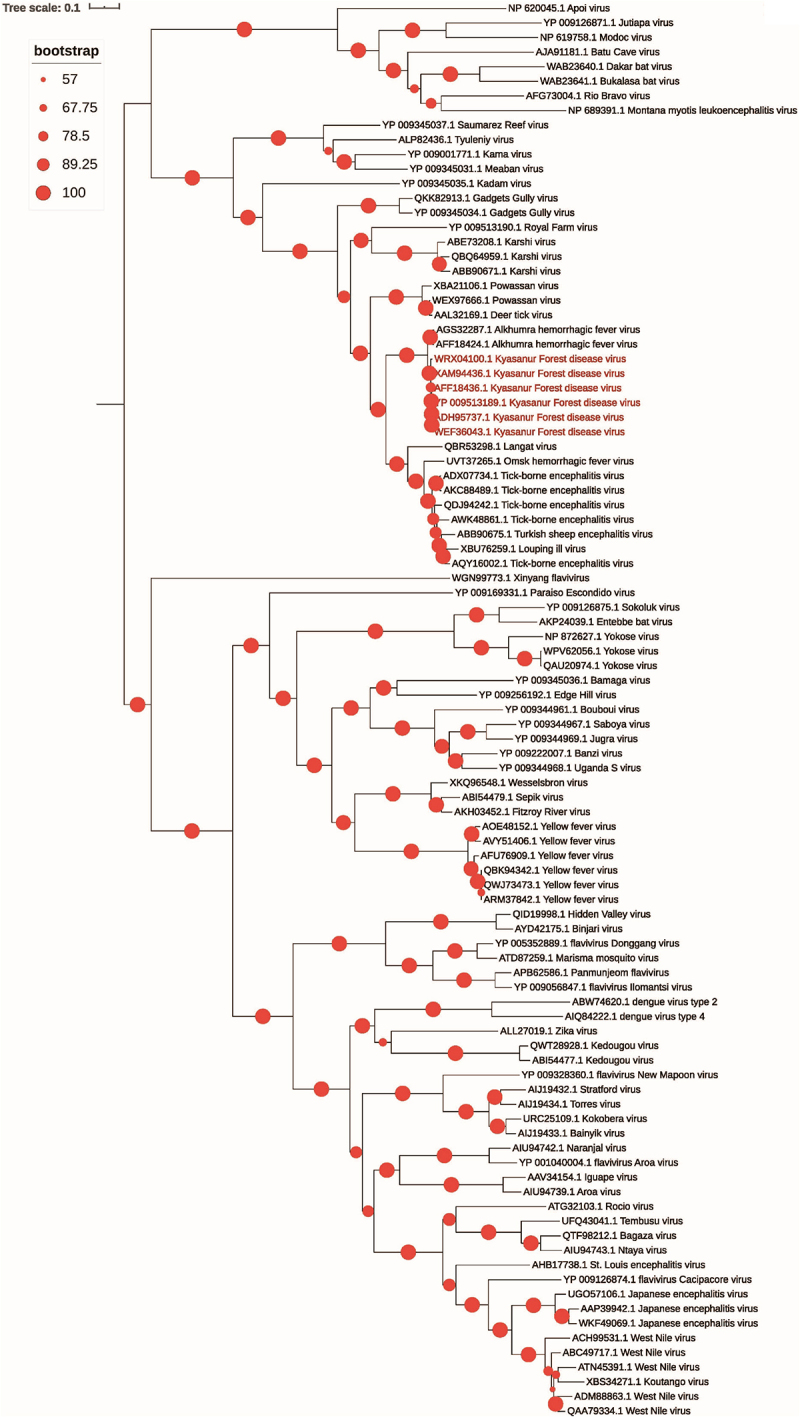
Phylogenetic relationship of KFDV with tick-borne flaviviruses based on polyprotein sequences. Maximum likelihood phylogenetic analysis using polyprotein sequences of KFD virus with other tick-borne flaviviruses. The KFDV sequences are highlighted in red color. Bootstrap support values, calculated using IQ-TREE, are displayed by circles to indicate the confidence levels of the branching patterns. The scale bar represents the number of estimated substitutions per site.

Phylogenetic and comparative genomic studies have consistently shown a close evolutionary relationship between the KFDV and the AHFV. Palanisamy *et al*. 2018 conducted an *in silico* comparative genome analysis that revealed significant sequence similarity between KFDV and AHFV [52]. This finding supports a shared ancestry and emphasizes the need for broader surveillance to uncover potential missing evolutionary links. In addition, Yadav *et al*. (2020) analyzed 48 complete KFDV genomes, including 46 newly sequenced strains, as well as 28 E-gene sequences spanning the years 1957 to 2017 [53]. Their study documented the spatiotemporal spread of the virus from Karnataka to Goa and Maharashtra, identified a genetically divergent subgroup of KFDV strains emerging around 1980, and provided evidence of adaptive evolution near the envelope protein dimer interface, which may have functional significance. Notably, they observed slightly higher evolutionary rates in the E-gene compared to the overall genomes, highlighting the limitations of relying on a single gene for phylogenetic analysis [53]. Supporting these findings, an updated maximum likelihood phylogenetic analysis, using publicly available amino acid sequences, based on 97 polyprotein sequences of KFDV, including other tick-borne flaviviruses, also positions KFDV as closely related to AHFV sequences (Figure 5). The KFDV polyproteins share ~96–97% sequence identity with AHFV, highlighting their close evolutionary relationship (Figure 5).

Despite several decades of genomic surveillance, no intermediate or more closely related virus has been identified, suggesting that ancestral or transitional lineages may remain unsampled or could no longer be circulating. This persistent clustering underscores the need for expanded field sampling and full-genome sequencing to better understand the evolutionary trajectory of tick-borne flaviviruses. Additionally, it is crucial to recognize that many available KFDV genome sequences, particularly those from older isolates, were obtained from passaged viral strains that underwent multiple rounds of adaptation in cell culture or animal models. Such passaging can introduce genetic artifacts that do not accurately reflect natural viral evolution. For instance, Sawatsky *et al*. 2014 demonstrated that repeated passaging of KFDV led to the loss of hemorrhagic properties in a mouse model, likely due to culture-induced mutations [54]. These findings highlight the necessity of interpreting existing sequence data with caution and emphasize the importance of prioritizing minimally passaged or field-derived virus isolates in future evolutionary studies.

## Transmission dynamics and reservoirs of KFDV

KFDV circulates primarily through an enzootic cycle involving ticks as vectors and wildlife reservoirs [2,22,55]. The principal vectors are ticks of the genus *Haemaphysalis*, with *H. spinigera* and *H. turturis* identified as key species in Kerala’s Malappuram and Wayanad districts [22]. However, other *Haemaphysalis* species, such as *H. formosensis*, *H. cuspidata*, and *H. bispinosa*, and genera like *Ixodes*, *Rhipicephalus*, and *Dermacentor*, also harbor and transmit the virus [56–58]. Ticks acquire KFDV during any life stage, with transstadial and transovarial transmission ensuring viral persistence across generations [59,60]. Recent mechanistic and spatial machine learning models have advanced understanding of KFDV transmission dynamics and improved risk prediction, highlighting the roles of transovarial transmission, small mammals, and birds in disease maintenance [34,35]. Humans can acquire ticks at any life stage – larva, nymph, or adult – and infected ticks have been reported not only in forest habitats but also in plantations, agricultural fields, and peri-domestic environments, as documented by Burthe et al. (2021) and the Monkey Fever Risk Project [36,60]. After feeding, infected nymphs molt into an adult. The adult then lays eggs that hatch into larvae. These larvae feed on small mammals or primates, perpetuating the cycle [61].

Wild primates, including red-faced bonnet monkeys (*Macaca radiata*) and black-faced langurs (*Semnopithecus entellus*), can act as amplifying hosts, developing high viremia that facilitates transmission to feeding ticks. However, recent evidence suggests that, despite their role in amplifying infection, primates may not be the primary hosts maintaining KFDV in the environment [35,36]. Their fatal febrile illness can cause postmortem detachment of infected ticks; however, such deaths likely signal wider regional virus activity rather than localized hotspots, and the risk to humans may extend far beyond the immediate site of monkey mortality [36]. Furthermore, recent field evidence indicates that transovarial transmission of KFDV may occur in wild tick populations [35,60], which could allow the virus to persist across tick generations without the need for an amplifying vertebrate host. This has important implications for understanding human disease risk and the year-round maintenance of infection in endemic areas. Detection of KFDV-neutralizing antibodies in buffaloes, cattle, wild boars, and bats could be due to cross-reactivity with other flaviviruses and should therefore be interpreted with caution and warrant further investigations [62].

**Figure 6.**
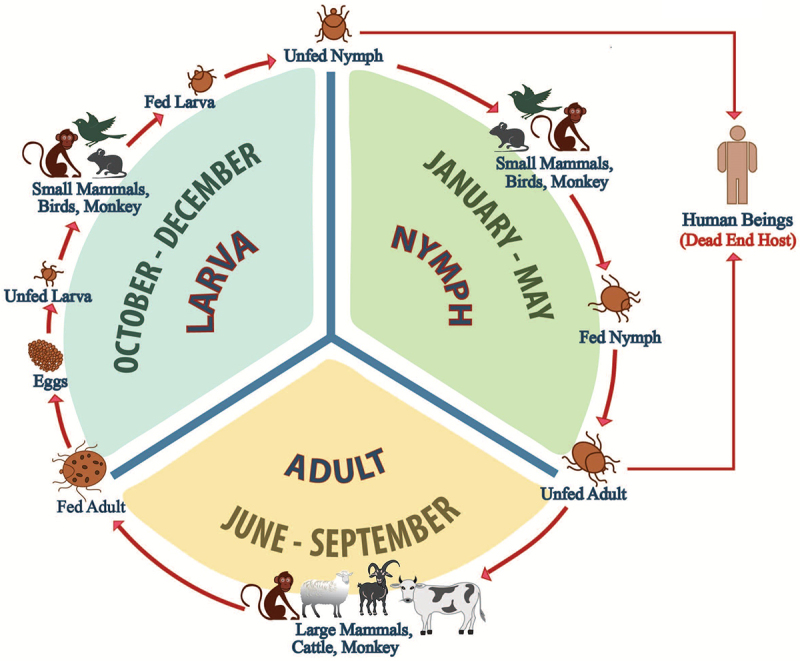
Life cycle of *Haemaphysalis* ticks and transmission dynamics of KFDV. The figure illustrates the developmental stages of *Haemaphysalis* ticks, including egg, larva, nymph, and adult, highlighting their role in KFDV maintenance and transmission.

Humans are dead-end hosts, acquiring infection through bites in areas where ticks occur – these may include not only forests but also pathways, plantations, rice paddies, gardens, and other vegetated sites frequented by animal hosts and people. No human-to-human transmission occurs, as infection is acquired exclusively through the bite of an infected tick. This spillover underscores the zoonotic nature of KFDV, highlighting the interconnected roles of forest ecosystems, wildlife reservoirs, and human behavior in disease dynamics, and reinforcing the need for One Health approaches [63]. The transmission cycle of the *Haemophysalis* tick is illustrated in Figure 6.

## Pathophysiology of KFDV

The pathophysiology of KFD is marked by systemic viral invasion, immune dysregulation, and multiorgan damage. Following transmission via infected tick bites, KFDV enters the bloodstream and targets monocyte-derived dendritic cells and vascular endothelial cells, where initial replication occurs [64,65]. This phase triggers high-level viremia, detectable as early as 36–48 hours before symptom onset, peaking within the first week of illness [1,47,66]. Viral dissemination to the liver, spleen, and lymph nodes amplifies tissue injury, driven by direct cytopathic effects and inflammatory responses [6].

Endothelial cell infection disrupts vascular integrity, increasing permeability and contributing to hemorrhagic manifestations [65]. Coagulopathy arises from multifactorial mechanisms, including disseminated intravascular coagulation (DIC), hepatic dysfunction, and bone marrow suppression, particularly megakaryocyte injury impairing platelet production [64]. DIC aggravates hemorrhage by depleting clotting factors and inducing plasma leakage, which can trigger hypovolemic shock or multiorgan failure in severe cases.

Hematological abnormalities are hallmark features. Leukopenia, characterized by lymphopenia and neutropenia (neutrophil counts < 2000 cells/ml), reflects bone marrow suppression and immune cell redistribution [30,66]. Thrombocytopenia further aggravates bleeding tendencies, manifesting as epistaxis, gingival bleeding, or gastrointestinal hemorrhage [66]. Hepatic involvement is evidenced by elevated transaminases, hypoalbuminemia, and hyperbilirubinemia, indicating hepatocellular injury and impaired synthetic function [1,6]. Postmortem animal studies reveal hepatic focal necrosis, renal tubular epithelial shedding, and splenic erythrophagocytosis, underscoring systemic pathology [30,55].

Severe cases progress to complications such as hemorrhagic pneumonitis, bronchopneumonia, encephalopathy, or coma, often preceding fatal outcomes [30]. Despite systemic inflammation, humans remain dead-end hosts, as the virus cannot sustain transmission between individuals. The interplay of viral tropism, immune-mediated damage, and coagulopathy defines KFD’s pathophysiology, highlighting the need for early clinical intervention to mitigate life-threatening sequelae.

In murine models, infection with KFDV results in pronounced morbidity and mortality, markedly higher than that observed with AHFV, a closely related tick-borne flavivirus [67]. The neuropathology associated with KFDV infection is characterized by widespread neuronal apoptosis, gliosis, and perivascular cuffing, particularly within the cerebrum and hippocampus [68,69]. These histopathological changes are accompanied by observable clinical signs, including motor neuron dysfunction and progressive hindquarter paralysis, suggestive of central nervous system involvement and neuroinvasion [69].

## Host immune responses to KFDV

The host immune response to KFDV involves a dynamic interplay between innate and adaptive mechanisms, though a comprehensive understanding remains limited. Early innate responses are initiated through viral recognition by infected cells, triggering type 1 interferons (IFN-α/β) via signaling cascades such as the JAK/STAT pathway, which induce an antiviral state [70]. Elevated IFN levels during the acute phase of KFD, as observed by Sathe et al. (1991), suggest a robust innate defense, though KFDV-specific studies are sparse [71].

**Figure 7.**
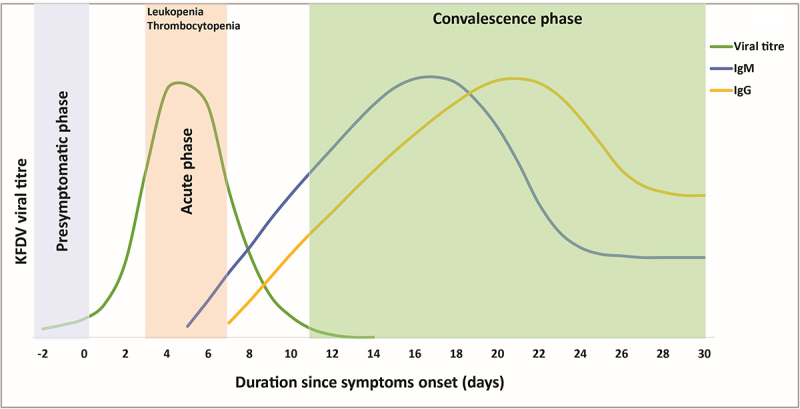
Dynamics of KFDV-specific IgM and IgG responses in relation to viremia during acute and convalescent phases of KFD. The figure depicts the progression of viremia and the corresponding host immune response following KFDV infection.

Humoral immunity plays a pivotal role in controlling viral infection. Complement-fixing and hemagglutination inhibition antibodies emerge early, followed by neutralizing antibodies (NAbs) targeting the viral E protein by the second week, peaking at three weeks [72]. These NAbs persist for over a decade, indicating durable immunological memory [72]. IgM antibodies appear by days 5–6 post-onset, followed by IgG around days 7–8, with IgG correlating strongly with viral RNA clearance [47,66]. Viremia peaks between days 3–6 and declines as IgG rises, underscoring the antibody-mediated control of infection [66]. The viral load and antibody kinetics during the acute and convalescent phases in KFD are shown in Figure 7. Recent studies confirm sustained anti-KFDV IgG levels in recovered individuals, regardless of vaccination status, suggesting natural infection induces lasting immunity comparable to vaccines [15].

Cellular immunity, particularly CD8^+^ T cell activation, is critical during acute infection. Devadiga et al. (2020) observed heightened CD8^+^ T cell responses coinciding with viral clearance, alongside robust B cell activity [66]. *Ex vivo* studies using KFDV antigens demonstrated stronger T cell proliferation in recovered individuals than controls, highlighting the generation of cellular immunity in KFDV-exposed individuals [15]. However, the licensed vaccine shows limited cellular immunity enhancement in recovered hosts, emphasizing the need for improved vaccine strategies [15].

Despite these advancements, further research is needed to elucidate the full spectrum of host immune responses to KFDV, including identifying protective immune factors and the mechanisms underlying viral clearance. Such studies could provide valuable insights for developing effective vaccines and therapeutic strategies against KFD.

## Development and challenges of the KFD vaccines

Efforts to develop a vaccine for KFD date back to the early 1960s, driven by the urgent need to contain this emerging tick-borne viral hemorrhagic fever endemic to parts of India. Initial vaccine strategies focused on exploiting antigenic similarities between KFDV and the Far-Eastern tick-borne encephalitis virus (TBEV-FE), previously known as Russian Spring-Summer encephalitis virus [73]. In collaboration with the ICMR, the Walter Reed Army Institute of Research, supported by the Rockefeller Foundation, formulated a formalin-inactivated TBEV-FE vaccine derived from mouse brains. However, this heterologous vaccine failed to confer protective immunity in human recipients, emphasizing cross-protection limitations among antigenically related flaviviruses [74–76].

Subsequent vaccine development shifted focus to the homologous KFDV. A series of experiments involved growing KFDV in Swiss albino mouse brains, followed by formalin inactivation. This preparation induced NAbs in mice, but its short shelf life rendered it unsuitable for large-scale immunization campaigns [77]. Another attempt employed chick embryo propagation of KFDV, but this formulation also failed to elicit protective immunity in animal models [78,79]. A live-attenuated vaccine candidate was also explored through serial passaging-two in chick embryo tissue culture and 169 in monkey kidney cell cultures – but it did not progress to clinical use, due to safety or immunogenicity concerns [80].

The most notable progress came in 1966, when a formalin-inactivated vaccine was formulated by propagating KFDV in chick embryo fibroblast cultures. This vaccine demonstrated adequate protective immunity, safety, and stability in preclinical trials [81,82]. Although this formulation became the first and only licensed KFD vaccine, its real-world effectiveness was modest. Field studies revealed a protective effectiveness of approximately 62% after two primary doses, increased to 83% with booster administration [83]. As a result, the vaccine was recommended for individuals aged 7 to 65 years in endemic regions, administered in a two-dose schedule spaced at least four weeks apart. To maintain protective immunity, booster doses are advised every 6–9 months, with annual boosters in areas where recent KFD cases are reported in humans or monkeys. Despite its availability, the long-used formalin-inactivated KFD vaccine faced significant challenges – its highly painful administration, requirement for multiple booster doses, and limited long-term protection contributed to low uptake and community reluctance. The eventual withdrawal of this vaccine due to poor efficacy may have further deepened mistrust toward vaccination in affected regions. Recent evidence, however, indicates that the TBEV vaccine offers cross-protection against KFDV with higher efficacy than the old formalin-based vaccine [84], presenting a promising alternative for future control efforts.

The development of more immunogenic and longer-lasting vaccines remains a critical unmet need. Modern approaches, such as recombinant subunit vaccines, vector-based platforms, and mRNA technologies, offer promise but have yet to be fully explored for KFD. Recent studies investigated a vesicular stomatitis virus (VSV)-based vaccine expressing KFDV M and E proteins, demonstrating protection in mice and non-human primates and also cross-reactivity against AHFV [85,86]. Additionally, efforts to develop more effective vaccines against KFDV have incorporated traditional and cutting-edge approaches. One such strategy under development involves a β-propiolactone-inactivated whole-virion vaccine, aiming to provide broader immune protection by preserving the structural integrity of viral proteins. Parallel to this, researchers investigate subunit vaccines based on the KFDV envelope protein, a critical antigen involved in host cell entry and a key target for neutralizing antibodies. Notably, advances in computational immunology have accelerated the rational design of vaccine candidates. An *in silico* study by Arumugam and Varamballi (2021) identified B-cell and T-cell epitopes from conserved regions of the envelope protein to construct multi-epitope vaccine constructs [87]. These constructs demonstrated promising immunogenicity profiles, including high binding affinity to toll-like receptor 2 (TLR2) and robust predicted immune responses. More recently, Dey *et al*. (2023) and Kasibhatla et al. (2024) expanded on this work using immune-informatics tools to identify additional conserved epitopes across KFDV structural and non-structural proteins, proposing multi-target strategies to elicit broad immunity [88–90]. While these computational approaches provide a valuable foundation, transitioning from *in silico* predictions to viable vaccine candidates requires rigorous preclinical and clinical validation to assess their safety, efficacy, and protective potential *in vivo*.

## Patient management

During the KFD transmission season, every patient presenting with fever in endemic areas is treated as a suspected KFD case. This approach enables early diagnosis and timely intervention, which are crucial in preventing complications. Upon laboratory confirmation, patients are admitted to the hospital for close monitoring of vital signs. Hospitalized patients typically receive intravenous fluids to address dehydration and undergo daily complete blood counts to monitor platelet levels, which inform referral decisions. Treatment also includes antipyretics and analgesics for fever and pain. In severe KFD cases, blood transfusions may be necessary [91]. Antimicrobial therapy is administered in cases of secondary bacterial infection, while anticonvulsants and corticosteroids are used to manage neurological complications [39–41]. Early diagnosis and prompt supportive care have significantly contributed to reducing KFD-related mortality in recent years.

## Antivirals

Currently, no approved antiviral drug has been developed explicitly for treating KFD. However, several compounds with activity against flaviviruses have been explored as potential therapeutic options. For instance, Z2 is a synthetic peptide that targets the flavivirus’s envelope protein and has demonstrated inhibitory effects against ZIKV, Dengue virus (DENV), and YFV *in vitro*. In animal models, including pregnant mice, Z2 significantly reduced viral load and prevented vertical transmission. Similarly, P5, a peptide derived from the stem region of the E protein, has shown efficacy against Japanese encephalitis virus (JEV) and ZIKV by blocking key conformational changes required for viral entry [92].

NITD008, an adenosine nucleoside analog, has exhibited broad-spectrum antiviral activity against several flaviviruses, including KFDV. Despite its promising *in vitro* efficacy, its further development was discontinued due to significant toxicity observed in preclinical studies [93,94]. Ivermectin, a widely used antiparasitic agent, has been found to inhibit the helicase activity of the NS3 protein, thereby suppressing replication of flaviviruses such as YFV, DENV, JEV, and West Nile virus (WNV) *in vitro*. While these findings are encouraging, additional studies are needed to confirm their antiviral potential in clinical settings [95].

Arbidol (Umifenovir), an antiviral approved for influenza treatment in China and Russia, has also been shown to inhibit WNV and ZIKV by preventing the interaction between virions and host cells [96]. Another repurposed compound, methotrexate (MTX), commonly used in oncology and autoimmune diseases, demonstrated anti-ZIKV activity by targeting dihydrofolate reductase, a key enzyme in nucleotide synthesis [97]. Gossypol, a naturally occurring polyphenolic compound, has shown inhibitory effects on ZIKV by binding to the envelope protein domain III. It also effectively suppressed all four DENV serotypes in cell cultures, highlighting its potential as a broad-spectrum antiviral candidate [98]. Additionally, ST-148, a small-molecule capsid inhibitor, has been shown to reduce cytopathic effects and replication of all four DENV serotypes *in vitro*, suggesting its potential as a protease-targeting antiviral [98].

A key challenge in antiviral drug development is the rapid emergence of drug-resistant viral variants. Viral infections trigger multiple intracellular signaling cascades that establish an antiviral state. Nevertheless, many viruses have evolved sophisticated strategies to hijack these pathways to support their replication and survival within the host [99–103]. This viral dependence on host signaling networks presents a potential opportunity for developing host-directed antiviral agents [104].

Unlike antiviral agents that target viral proteins directly, drugs that interfere with essential host cell processes typically exhibit a reduced likelihood of inducing resistance [101,102,105–110]. In addition, many cellular factors are commonly exploited by multiple viruses or across numerous family members. Therefore, host-directed antiviral agents usually exhibit broad-spectrum antiviral activity [100,111,112].

A significant limitation in combating KFDV is our incomplete understanding of how the virus engages and manipulates host cell signaling pathways. Further research into these interactions could open up new avenues for the design of more effective antiviral interventions.

## Preventive measures for KFD: A multifaceted approach

KFD remains a public health challenge due to a lack of specific antivirals and an effective vaccine, necessitating reliance on multifaceted preventive strategies. Enhanced surveillance systems incorporating geospatial mapping [10] and mobile app-based reporting under IDSP supported real-time tracking of monkey deaths and human cases, which is important for the containment of the early outbreak. Community engagement through local leaders and AI-enabled digital platforms fosters awareness about tick biology, KFD transmission, and prompt reporting. Recent studies have advanced predictive modeling for KFD. Purse et al. (2020) developed the KFD Explorer tool, which integrates ecological, environmental, and epidemiological data to identify high-risk areas and provide real-time alerts. In Karnataka, it is actively used to guide surveillance, target interventions, and strengthen outbreak preparedness [34].

The One Health approach, inherently interdisciplinary – encompassing ecological, veterinary, medical, and social sciences – recognizes the interconnectedness of human, animal, and environmental health, and bridges these sectors to disrupt zoonotic cycles [113]. In one instance, the One Health approach has demonstrated promising results in containing and preventing KFDV outbreaks. In Wayanad, Kerala, this approach effectively managed the 2015 epidemic through multisectoral collaboration, integrated surveillance, and stakeholder meetings, and will help institutionalize the strategy at the ministerial level [114]. Co-producing research and tools with stakeholders through One Health approaches in KFD-endemic regions such as Wayanad, Kerala, and Shimoga, Karnataka, has yielded evidence-based interventions, including educational campaigns and predictive mapping. These initiatives have also generated web-based decision support systems and educational materials for communities and health workers, crucial for predicting disease risk and guiding effective interventions in tropical settings [115]. Studies show that infected ticks can bite people not only in forests but also at forest edges, on farmland, and around homes. The risk is highest in areas where farmland and forested land are close together or overlap [115]. In the endemic areas, socially vulnerable groups face the most significant risk and impact from KFDV. ICMR has employed a One Health focus to address KFDV, emphasizing early detection, diagnostic tool development, stakeholder collaboration, and capacity building to understand and control the disease’s spread [116].

Ticks are vectors of numerous zoonotic diseases and are second only to mosquitoes as disease transmitters [117]. Tick-borne diseases considerably hamper animal productivity and lead to significant morbidity and mortality in both animals and humans. Targeted tick control employs acaricides like benzene hexachloride [7]. However, acaricide resistance in ticks poses a significant challenge to controlling tick-borne diseases, including KFD in endemic regions. The overuse of acaricides has led to the development of resistant tick populations, limiting the efficacy of existing control methods [118]. However, modern environmental concerns have spurred interest in alternative, eco-friendly biopesticides and integrated pest management systems. In India, ticks are vectors of various zoonotic pathogens, including the KFD virus, necessitating reassessment of tick control strategies [117].

Personal protection is strengthened by permethrin-treated clothing and DEET-based repellents, reducing bite risks in endemic forests [119]. A thorough check of bodies for ticks after visiting a forested area is recommended [7,14,23]. The Monkey Fever Risk Project has developed targeted, co-produced educational materials and guidance, now being distributed across multiple KFD-affected states, and accessible via its online platform (https://monkeyfeverrisk.ceh.ac.uk/decision-support-tools-and-risk-guidance). Capacity building through training of the healthcare workers and intersectoral collaboration further strengthens outbreak response [49].

## Future perspectives

As KFD continues to emerge and reemerge across an expanding geographical range in India, addressing its growing public health threat demands a forward-looking, integrated strategy grounded in scientific innovation, ecological understanding, and public health preparedness. The geographic expansion of KFD beyond its original foci in Karnataka into Tamil Nadu, Kerala, Goa, and Maharashtra underscores the dynamic interplay between environmental change, deforestation, and human encroachment into wildlife habitats [31,32]. Future efforts must prioritize surveillance systems that are real-time and geospatially enabled and integrated across human, animal, and ecological health domains – embodying the One Health framework [114,116].

The current urgency is the development of an effective KFD vaccine, as the licensed vaccine offers only limited protection and needs frequent booster doses, limiting its effectiveness and acceptance [83]. Recent studies show that individuals recovered from natural infection of KFDV acquire long-term immunity without reinfection, highlighting the importance of investigating protective correlates in recovered cases [15]. Immunological studies employing high-throughput technologies such as transcriptomics, proteomics, and single-cell profiling could help identify immune signatures critical for designing durable, next-generation vaccines. Computational immunology and reverse vaccinology are also paving the way for rational vaccine design – multi-epitope vaccine candidates based on conserved regions of the KFDV envelope and non-structural proteins have shown promise *in silico* [87–89], but require experimental validation in preclinical and clinical studies.

In parallel, the development of antivirals against KFDV remains a pressing need. Given the lack of specific therapeutics, repurposing broad-spectrum antivirals that target flaviviral proteins (such as NS3 helicase and NS5 polymerase) or host-targeted pathways holds promise. Compounds such as NITD008, ivermectin, arbidol, and gossypol derivatives have demonstrated anti-flaviviral activity *in vitro* and may serve as scaffolds for KFDV-targeted therapeutics [95,98,104]. Moreover, host-directed antiviral agents that modulate conserved signaling pathways (e.g. MAPK, ROCK, MNK1) are increasingly viewed as robust options due to their broad-spectrum potential and lower risk of resistance [101,112].

Improved diagnostics are equally essential. While RT-PCR and ELISA remain the primary diagnostic assays, their dependence on laboratory infrastructure limits field applicability. Therefore, future research should focus on developing lateral flow immunoassays and LAMP-based POCTs that are rapid, low-cost, and suitable for use in remote healthcare settings. The Truenat KFD assay represents a successful prototype of such field-deployable molecular diagnostics, but expanding its reach and complementing it with serological POCTs will be crucial [50].

Another important frontier is the molecular epidemiology and evolution of KFDV. Despite high sequence conservation among isolates, phylogenetic analyses suggest possible recombination events and unexplored genetic diversity in under-surveyed regions [51,120]. Expanding full-genome surveillance beyond Karnataka to newly affected and neighboring states is critical to detect emerging variants and assess viral evolution in response to ecological pressures. Such efforts will also aid in monitoring vaccine efficacy and developing strain-adapted immunogens.

Predictive risk mapping, developed through interdisciplinary collaboration, has proven helpful for spatially targeting KFD interventions, particularly in Karnataka [34]. However, further research is needed to determine whether the same ecological and human exposure factors apply in other regions, which may have different drivers of disease risk. Recent advances have enhanced predictive capabilities by integrating event-based surveillance data – such as news reports and internet search trends – with weather variables. Machine learning models like Extreme Gradient Boosting and Long Short-Term Memory have demonstrated improved performance in forecasting KFD outbreaks [121]. Integrating these predictive tools with digital health reporting systems could revolutionize early warning and rapid response systems in the endemic zones.

## Data Availability

Data sharing not applicable to this article as no new data were created or analyzed in this study.

## References

[1] Work TH, Trapido H, Murthy DP, et al. Kyasanur forest disease. iii. A preliminary report on the nature of the infection and clinical manifestations in human beings. Indian J Med Sci. 1957;11:619–18. 13474777

[2] Varma M, Trapido H, Rajagopalan P. Studies on ticks as possible vectors of Kyasanur forest disease. Public Heal Med Sci. 1957;17:88–90.

[3] Lakshmana R, Rao RL. Clinical observations on Kyasanur forest disease cases. J Indian Med Assoc. 1958;31:113–116. 13588005

[4] Gurav YK, Yadav PD, Gokhale MD, Chiplunkar TR, Vishwanathan R, Patil DY, et al. Kyasanur forest disease prevalence in Western Ghats proven and confirmed by recent outbreak in Maharashtra, India, 2016. Vector-Borne Zoonotic Dis. 2018;18(3):164–172. doi: 10.1089/vbz.2017.2129 29336707

[5] Upadhyaya S, Dpnn M, Anderson CR. Kyasanur forest disease in the human population of Shimoga District, Mysore state, 1959–1966. Indian J Med Res. 1975;63:1556–1563. 1222964

[6] Pavri K. Clinical, Clinicopathologic, and hematologic features of Kyasanur forest disease. Rev Infect Dis. 1989;11(Supplement_4):S854–9. doi: 10.1093/clinids/11.Supplement_4.S854 2665018

[7] Banerjee K. Kyasanur forest disease. Arboviruses Epidemiol Ecol. 1992:III 94–111.

[8] Holbrook MR. Kyasanur forest disease. Antiviral Res. 2012;96(3):353–362. doi: 10.1016/j.antiviral.2012.10.005 23110991 PMC3513490

[9] Munivenkatappa A, Yadav PD, Sahay RR, et al. Clinical, epidemiological, and molecular investigation of Kyasanur forest disease from Karnataka state, India during 2018–2019. Infect Dis (Auckl). 2024;56(2):145–156. doi: 10.1080/23744235.2023.2282042 37966909

[10] Murhekar MV, Kasabi GS, Mehendale SM, et al. On the transmission pattern of Kyasanur forest disease (KFD) in India. Infect Dis Poverty. 2015;4:37. doi: 10.1186/s40249-015-0066-9 26286631 PMC4545326

[11] Tandale BV, Balakrishnan A, Yadav PD, et al. New focus of Kyasanur forest disease virus activity in a tribal area in Kerala, India, 2014. Infect Dis Poverty. 2015;4:10–13. doi: 10.1186/s40249-015-0044-2 25750730 PMC4351674

[12] Patil DY, Yadav PD, Shete AM, et al. Occupational exposure of cashew nut workers to Kyasanur forest disease in Goa, India. Int J Infect Dis. 2017;61:67–69. doi: 10.1016/j.ijid.2017.06.004 28627428

[13] Mourya DT, Yadav PD, Sandhya VK, et al. Spread of Kyasanur forest disease, Bandipur tiger reserve, India, 2012–2013. Emerg Infect Dis. 2013;19(9):1540–1541. doi: 10.3201/eid1909.121884 23977946 PMC3810911

[14] Awate P, Yadav P, Patil D, et al. Outbreak of Kyasanur forest disease (monkey fever) in Sindhudurg, Maharashtra state, India, 2016. J Infect. 2016;72(6):759–761. doi: 10.1016/j.jinf.2016.03.006 26997635

[15] Kaushal H, Kartaskar RS, Chiplunkar T, et al. Cellular immune responses against γ-inactivated antigen in the recovered cases of Kyasanur forest disease. Viral Immunol. 2024;37:101–106. 38315741 10.1089/vim.2023.0107

[16] Trapido H, Galindo P. The epidemiology of yellow fever in Middle America. Exp Parasitol. 1956;5(3):285–323. doi: 10.1016/0014-4894(56)90041-8 13317949

[17] Drummond RO, Rajagopalan PK, Sreenivasan MA, et al. Tests with ixodicides for the control of the tick vectors of Kyasanur forest disease. J Med Ent. 1969;6(3):245–251. doi: 10.1093/jmedent/6.3.245 5820842

[18] Work TH. Russian spring-summer virus in India: Kyasanur forest disease. Prog Med Virol. 1958;1:248–277. 13579010

[19] Casals J, Brown LV. Hemagglutination with arthropod-borne viruses. J Exp Med. 1954;99(5):429–449. doi: 10.1084/jem.99.5.429 13163320 PMC2136253

[20] Work TH, Trapido H. Kyasanur forest disease: a new infection of man and monkeys in tropical India by a virus of the Russian spring-summer complex. Public Heal Med Sci. 1957;17:80–84.

[21] Directorate of Health and Family Welfare Services, Government of Karnataka. Operational Manual Kyasanur Forest Disease. 2020.

[22] Sadanandane C, Elango A, Marja N, et al. An outbreak of Kyasanur forest disease in the Wayanad and Malappuram districts of Kerala, India. Ticks Tick Borne Dis. 2017;8(1):25–30. doi: 10.1016/j.ttbdis.2016.09.010 27692988

[23] Bhatia B, Feldmann H, Marzi A. Kyasanur forest disease and alkhurma hemorrhagic fever virus—two neglected zoonotic pathogens. Microorganisms. 2020;8(9):1–17. doi: 10.3390/microorganisms8091406 PMC7564883 32932653

[24] Integrated Disease Surveillance Programme (IDSP). Weekly outbreaks. n.d [cited 2025 Feb 17]. Available from: https://idsp.mohfw.gov.in/index4.php?lang=1&level=0&linkid=406&lid=3689

[25] National Health Mission. Integrated Disease Surveillance Programme. Disease control Programmes/integrated+Disease+Surveillance+Programme+(IDSP)/en. n.d [cited 2025 Feb 18]. Available from: https://nhm.karnataka.gov.in/page/NHM+COMPONENTS/Communicable

[26] Commissionerate of Health & Family Welfare Services. Weekly Infectious disease Report - Commissionerate of health & family welfare services. n.d [cited 2025 Feb 18]. Available from: https://hfwcom.karnataka.gov.in/info-4/Weekly+Infectious+Disease+Report/en

[27] Sarkar JK, Chatterjee SN. Survey of antibodies against arthropod-borne viruses in the human sera. Indian J Med Res. 1962;50:833–841. 13991507

[28] Mourya DT, Yadav PD, Mehla R, et al. Diagnosis of Kyasanur forest disease by nested RT-PCR, real-time RT-PCR and IgM capture elisa. J Virol Methods. 2012;186(1–2):49–54. doi: 10.1016/j.jviromet.2012.07.019 22874757

[29] Padbidri VS, Wairagkar NS, Joshi GD, et al. A serological survey of arboviral diseases among the human population of the Andaman and Nicobar Islands, India. Southeast Asian J Trop Med Public Health. 2002;33:794–800. 12757228

[30] Pattnaik P. Kyasanur forest disease: an epidemiological view in India. Rev Med Virol. 2006;18:211. 10.1002/rmv.495 16710839

[31] Ajesh K, Nagaraja BK, Sreejith K. Kyasanur forest disease virus breaking the endemic barrier: an investigation into ecological effects on disease emergence and future outlook. Zoonoses Public Health. 2017;64(7):e73–80. doi: 10.1111/zph.12349 28220635

[32] Chakraborty S, Sander WE, Allan BF, et al. Retrospective study of Kyasanur forest disease and deaths among nonhuman primates, India, 1957–2020. Emerg Infect Dis. 2021;27(7):1969–1973. doi: 10.3201/eid2707.210463 34152964 PMC8237885

[33] Walsh MG, Mor SM, Maity H, et al. Forest loss shapes the landscape suitability of Kyasanur forest disease in the biodiversity hotspots of the Western Ghats, India. Int J Epidemiol. 2019;48:1804–1814. doi: 10.1093/ije/dyz232 31740967

[34] Purse BV, Darshan N, Kasabi GS, et al. Predicting disease risk areas through co-production of spatial models: the example of Kyasanur forest disease in india’s forest landscapes. PLOS Negl Trop Dis. 2020;14:1–27. doi: 10.1371/journal.pntd.0008179 PMC7164675 32255797

[35] Hassall RMJ, Burthe SJ, Schäfer SM, et al. Using mechanistic models to highlight research priorities for tick-borne zoonotic diseases: improving our understanding of the ecology and maintenance of Kyasanur forest disease in India. PLOS Negl Trop Dis. 2023;17:e0011300. doi: 10.1371/journal.pntd.0011300 37126514 PMC10174626

[36] Burthe SJ, Schäfer SM, Asaaga FA, et al. Reviewing the ecological evidence base for management of emerging tropical zoonoses: Kyasanur forest disease in India as a case study. PLOS Negl Trop Dis. 2021;15:1–26. doi: 10.1371/journal.pntd.0009243 PMC8016103 33793560

[37] Iyer CG, Laxmana Rao R, Work TH, et al. Pathological findings in three fatal human cases of Kyasanur forest disease. Indian J Med Sci. 1959;13:1011–1022. 14406181

[38] Rajaiah P. Kyasanur forest disease in India: innovative options for intervention. Hum Vaccines Immunother. 2019;15(10):2243–2248. doi: 10.1080/21645515.2019.1602431 PMC6816375 30945970

[39] Adhikari Prabha MRM, Raghuveer CV, Bai Mam M. Clinical study of 100 cases of Kyasanur forest disease with clinicopathological correlation. Indian J Med Sci. 1993 May;47(5):124–130. 8225455

[40] Gupta N, Varma M, Saravu K. Difference in clinical presentation between the first and second phases of Kyasanur forest disease: an experience from a teaching hospital in south India. Infez Med. 2020;28:597–602. 33257636

[41] Wadia RS. Neurological involvement in Kyasanur forest disease. Neurol India. 1975;23:115–120. 1214955

[42] Gupta N, Chunduru K, Safeer KM, et al. Clinical and laboratory profile of patients with Kyasanur forest disease: a single-centre study of 192 patients from Karnataka, India. J Clin Virol. 2021;135:104735. doi: 10.1016/j.jcv.2021.104735 33493988

[43] Gladson V, Mathew S, B J, et al. A case series on the spectrum of complications observed in Kyasanur forest disease. Cureus. 2024;16:e59971. 38854314 10.7759/cureus.59971 PMC11162157

[44] Kuchelkar SP, Dias A, Gomes E, et al. Study of the clinical manifestations and risk factors in people affected during the first Kyasanur forest disease outbreak in Goa, India: a mixed method study. J Vector Borne Dis. 2024;61:23–28. doi: 10.4103/0972-9062.383650 38648403

[45] Munivenkatappa A, Sahay RR, Yadav PD, et al. Clinical & epidemiological significance of Kyasanur forest disease. Indian J Med Res. 2018;148(2):145–150. doi: 10.4103/ijmr.IJMR_688_17 30381537 PMC6206778

[46] Bhatt PN, Work TH, Varma MG, et al. Kyasanur forest diseases. iv. Isolation of Kyasanur forest disease virus from infected humans and monkeys of Shimogadistrict, Mysore state. Indian J Med Sci. 1966;20:316–320. 4957898

[47] Yadav P, Gurav Y, Shete A, et al. Kinetics of viral rna, immunoglobulin-M & G antibodies in Kyasanur forest disease. Indian J Med Res. 2019;150(2):186–193. doi: 10.4103/ijmr.IJMR_1929_17 31670274 PMC6829781

[48] Gupta N, Wilson W, Neumayr A, et al. Kyasanur forest disease: a state-of-the-art review. QJM An Int J Med. 2022;115(6):351–358. doi: 10.1093/qjmed/hcaa310 33196834

[49] Kaushal H, Das S, Kartaskar RS, et al. Kyasanur forest disease: a neglected zoonotic disease of India. In: Bhukya PL, Mhaske S, Sonkar CS, editors. Emerging Human Viral Diseases Volume I Respiratory Haemorrhagic Fever. Vol. 1. Springer Nature; 2023. p 401–417.

[50] Majumdar T, Shete A, Yadav P, et al. Point of care real-time polymerase chain reaction-based diagnostic for Kyasanur forest disease. Int J Infect Dis. 2021;108:226–230. doi: 10.1016/j.ijid.2021.05.036 34023493

[51] Dodd KA, Bird BH, Khristova ML, et al. Ancient ancestry of KFDV and AHFV revealed by complete genome analyses of viruses isolated from ticks and mammalian hosts. PLOS Negl Trop Dis. 2011;5:1–7. doi: 10.1371/journal.pntd.0001352 PMC3186760 21991403

[52] Palanisamy N, Akaberi D, Lennerstrand J, et al. Comparative genome analysis of alkhumra hemorrhagic fever virus with Kyasanur forest disease and tick-borne encephalitis viruses by the in silico approach. Pathog Glob Health. 2018;112(4):210–226. doi: 10.1080/20477724.2018.1471187 29745301 PMC6151960

[53] Yadav PD, Patil S, Jadhav SM, et al. Phylogeography of Kyasanur forest disease virus in India (1957–2017) reveals evolution and spread in the Western Ghats region. Sci Rep. 2020;10:1–12. doi: 10.1038/s41598-020-58242-w 32029759 PMC7005018

[54] Sawatsky B, Aj M, Holbrook MR, et al. Comparative pathogenesis of Alkhumra hemorrhagic fever and Kyasanur forest disease viruses in a mouse Model. PLOS Negl Trop Dis. 2014;8(6):e2934. doi: 10.1371/journal.pntd.0002934 24922308 PMC4055546

[55] Work TH, Roderiguez FR, Bhatt PN. Virological epidemiology of the 1958 epidemic of Kyasanur Forest disease. Am J Public Heal Nations Heal. 1959;49(7):869–874. doi: 10.2105/AJPH.49.7.869 PMC1372906 13661478

[56] Singh KR, Pavri KM, Anderson CR. Transmission of Kyasanur forest disease virus by Haemaphysalis. Indian J Med Res. 1964;52:566–573. 14184087

[57] Rajagopalan PK, Paul SD, Sreenivasan MA. Isolation of Kyasanur forest disease virus from the insectivorous bat, rhinolophus rouxi and from Ornithodoros ticks. Indian J Med Res. 1969;57:805–808. 5820428

[58] Shah SZ, Jabbar B, Ahmed N, et al. Epidemiology, pathogenesis, and control of a tick-borne disease- Kyasanur forest disease: current status and future directions. Front Cell Infect Microbiol. 2018;8:149. doi: 10.3389/fcimb.2018.00149 29868505 PMC5954086

[59] Pattnaik P. Kyasanur forest disease: an epidemiological view in India. Rev Med Virol. 2006;16(3):151–165. doi: 10.1002/rmv.495 16710839

[60] Burthe SJ, Kumbar B, Schäfer SM, et al. First evidence of transovarial transmission of Kyasanur forest disease virus in Haemaphysalis and rhipicephalus ticks in the wild. Parasites Vectors. 2025;18(1):14. doi: 10.1186/s13071-024-06643-5 39825388 PMC11740564

[61] Mourya DT, Patil DY, Yadav PD, et al. Expediency of dengue illness classification: the Sri Lankan perspective highly infectious tick-borne viral diseases: Kyasanur forest disease and crimean-congo haemorrhagic fever in India. WHO South-East Asia J Public Heal. 2014;3(1):8. doi: 10.4103/2224-3151.206890 28607249

[62] Bhat HR, Sreenivasan MA, Goverdhan MK, et al. Antibodies to Kyasanur forest disease virus in bats in the epizootic-epidemic area and neighbourhood. Indian J Med Res. 1978;68:387–392. 217820

[63] Asaaga FA, Young JC, Srinivas PN, et al. Co-production of knowledge as part of a OneHealth approach to better control zoonotic diseases. PLOS Glob Public Heal. 2022;2:e0000075. 10.1371/journal.pgph.0000075 PMC10021618 36962247

[64] Zapata JC, Cox D, Salvato MS. The role of platelets in the pathogenesis of viral hemorrhagic fevers. PLOS Negl Trop Dis. 2014;8(6):e2858. doi: 10.1371/journal.pntd.0002858 24921924 PMC4055450

[65] Sirmarova J, Salat J, Palus M, et al. Kyasanur forest disease virus infection activates human vascular endothelial cells and monocyte-derived dendritic cells. Emerg Microbes Infect. 2018;7(1):1–12. doi: 10.1038/s41426-018-0177-z 30401896 PMC6220120

[66] Devadiga S, Ak M, Prabhu SG, et al. Dynamics of human B and T cell adaptive immune responses to Kyasanur forest disease virus infection. Sci Rep. 2020;10(1):1–9. doi: 10.1038/s41598-020-72205-1 32943687 PMC7499197

[67] Dodd KA, Bird BH, Mebb J, et al. Kyasanur forest disease virus infection in mice is associated with higher morbidity and mortality than infection with the closely related alkhurma hemorrhagic fever virus. PLOS ONE. 2014;9(6):e100301. doi: 10.1371/journal.pone.0100301 24950196 PMC4065072

[68] Basu A, Yadav P, Prasad S, et al. An early passage human isolate of Kyasanur forest disease virus shows acute neuropathology in experimentally infected CD-1 mice. Vector-Borne Zoonotic Dis. 2016;16(7):496–498. doi: 10.1089/vbz.2015.1917 27171207

[69] Srikanth UK, Marinaik CB, Rao S, et al. Studies on the sequential pathology of Kyasanur forest disease (KFD) in mouse brain: KFD virus induces apoptosis of neurons in cerebrum and hippocampus. PLOS ONE. 2024;19:e0297143. doi: 10.1371/journal.pone.0297143 38427645 PMC10906829

[70] de la Fuente J, Antunes S, Bonnet S, et al. Tick-pathogen interactions and Vector competence: identification of molecular drivers for tick-borne diseases. Front Cell Infect Microbiol. 2017;7. doi: 10.3389/fcimb.2017.00114 PMC5383669 28439499

[71] Sathe PS, Dandawate CN, Sharadamma K, et al. Circulating interferon-alpha in patients with Kyasanur forest disease. Indian J Med Res. 1991;93:199–201. 1959947

[72] Achar TR, Patil AP, Jayadevaiah MS, et al. Persistance of humoral immunity in Kyasanur forest disease. Indian J Med Res. 1981;73:1–3. 6263796

[73] Gritsun TS, Frolova TV, Zhankov AI, et al. Characterization of a Siberian virus isolated from a patient with progressive chronic tick-borne encephalitis. J Virol. 2003;77:25–36. doi: 10.1128/JVI.77.1.25-36.2003 12477807 PMC140615

[74] Aniker SP, Work TH, Chandrasekharaiya T, et al. The administration of formalin-inactivated rsse virus vaccine in the Kyasanur forest disease area of Shimoga District, Mysore state. Indian J Med Res. 1962;50:147–152. 13861642

[75] Pavri K, Gokhale T, Shah K. Serological response to Russian spring-summar encephalitis virus vaccine as measured with Kyasanur forest disease virus. Indian J Med Res. 1962;50:153–161. 14484654

[76] Shah KV, Aniker S, Narashimha Murthy DP, et al. Evaluation of the field experience with formalin-inactivated mouse brain vaccine of Russian spring-summaer encephalitis virus against Kyasanur forest disease. Indian J Med Res. 1962;50:162–174. 13911122

[77] Mansharamani HJ, Dandawate CN, Krishnamurthy BG, et al. Experimental vaccine against Kyasanur forest disease (KFD) virus from mouse brain source. Indian J Pathol Bacteriol. 1965;12:159–177. 14340436

[78] Dandawate CN, Mansharamani HJ, Jhala HI. Experimental vaccine against Kyasanur forest disease (KFD) virus from embryonated eggs. I. Adaptation of the virus to developing chick embryo and preparation of formolised vaccines. Indian J Pathol Bacteriol. 1965;8:241–260. 5850010

[79] Dandawate CN, Mansharamani HJ, Jhala HI. Experimental vaccine against Kyasanur forest disease (KFD) virus from embryonated eggs. ii. Comparative immunogenicity of vaccines inactivated with formalin and beta-propio-lactone (bpl). Indian J Pathol Bacteriol. 1965;8:261–270. 5850011

[80] Bhatt PN, Anderson CR. Attenuation of a strain of Kyasanur forest disease virus for mice. Indian J Med Res. 1971;59:199–205. 5579236

[81] Mansharamani HJ, Dandawate CN, Mansharamani HJ. Experimental vaccine against Kyasanur forest disease (KFD) virus from tissue culture source. ii. Safety testing of the vaccine in cortisone sensitized Swiss albino mice. Indian J Pathol Bacteriol. 1967;10:25–32. 6036490

[82] Mansharamani HJ, Kb DC, Mansharamani HJ, et al. Experimental vaccine against Kyasanur forest disease (KFD) virus from tissue culture source. I. Some data on the preparation and antigenicity tests of vaccines. Indian J Pathol Bacteriol. 1967;10:9–24. 6068256

[83] Kasabi GS, Murhekar MV, Sandhya VK, et al. Coverage and effectiveness of Kyasanur forest disease (KFD) vaccine in Karnataka, South India, 2005–10. PLOS Negl Trop Dis. 2013;7(1):13–16. doi: 10.1371/journal.pntd.0002025 PMC3554520 23359421

[84] McAuley AJ, Sawatsky B, Ksiazek T, et al. Cross-neutralisation of viruses of the tick-borne encephalitis complex following tick-borne encephalitis vaccination and/or infection. NPJ Vaccines. 2017;2. doi: 10.1038/s41541-017-0009-5 PMC5627269 29263866

[85] Bhatia B, Meade-White K, Haddock E, et al. A live-attenuated viral vector vaccine protects mice against lethal challenge with Kyasanur forest disease virus. NPJ Vaccines. 2021;6:1–11. doi: 10.1038/s41541-021-00416-2 34907224 PMC8671490

[86] Bhatia B, Tang-Huau TL, Feldmann F, et al. Single-dose VSV-based vaccine protects against Kyasanur forest disease in nonhuman primates. Sci Adv. 2023;9(36):eadj1428. doi: 10.1126/sciadv.adj1428 37672587 PMC10482351

[87] Arumugam S, Varamballi P. In-silico design of envelope based multi-epitope vaccine candidate against Kyasanur forest disease virus. Sci Rep. 2021;11(1):11. doi: 10.1038/s41598-021-94488-8 34429443 PMC8384868

[88] Dey S, Pratibha M, Singh Dagur H, et al. Characterization of host receptor interaction with envelop protein of Kyasanur forest disease virus and predicting suitable epitopes for vaccine candidate. J Biomol Struct Dyn. 2024;42(8):4110–4120. doi: 10.1080/07391102.2023.2218924 37272880

[89] Kasibhatla SM, Rajan L, Shete A, et al. Construction of an immunoinformatics-based multi-epitope vaccine candidate targeting Kyasanur forest disease virus. PeerJ. 2025;13:e18982. doi: 10.7717/peerj.18982 40130172 PMC11932114

[90] Adla D, Josyula JVN, Ancha T, et al. Immunoinformatic based multi-epitope vaccine design and validation against Kyasanur forest disease: a tick borne viral infection. J Vector Borne Dis. 2025;62(3):369–379. doi: 10.4103/jvbd.jvbd_84_24 39936178

[91] John JK. Kyasanur forest disease: a status update. Adv Anim Vet Sci. 2014;2(6):329–336. doi: 10.14737/journal.aavs/2014/2.6.329.336

[92] Schmidt AG, Yang PL, Harrison SC. Peptide inhibitors of flavivirus entry derived from the E protein stem. J Virol. 2010;84:12549–12554. doi: 10.1128/JVI.01440-10 20881042 PMC3004314

[93] Xie X, Wang Q-Y, Xu HY, et al. Inhibition of dengue virus by targeting viral NS4B protein. J Virol. 2011;85:11183–11195. doi: 10.1128/JVI.05468-11 21865382 PMC3194949

[94] Nelson J, Roe K, Orillo B, et al. Combined treatment of adenosine nucleoside inhibitor NITD008 and histone deacetylase inhibitor vorinostat represents an immunotherapy strategy to ameliorate West Nile virus infection. Antiviral Res. 2015;122:39–45. doi: 10.1016/j.antiviral.2015.07.008 26225754 PMC4853296

[95] Mastrangelo E, Pezzullo M, De Burghgraeve T, et al. Ivermectin is a potent inhibitor of flavivirus replication specifically targeting NS3 helicase activity: new prospects for an old drug. J Antimicrob Chemother. 2012;67(8):1884–1894. doi: 10.1093/jac/dks147 22535622 PMC3888155

[96] Brooks MJ, Burtseva EI, Ellery PJ, et al. Antiviral activity of arbidol, a broad-spectrum drug for use against respiratory viruses, varies according to test conditions. J Med Virol. 2012;84(1):170–181. doi: 10.1002/jmv.22234 22028179

[97] Beck S, Zhu Z, Oliveira MF, et al. Mechanism of action of methotrexate against zika virus. Viruses. 2019;11:338. doi: 10.3390/v11040338 30974762 PMC6521145

[98] Gao Y, Tai W, Wang X, et al. A gossypol derivative effectively protects against Zika and dengue virus infection without toxicity. BMC Biol. 2022;20:1–21. doi: 10.1186/s12915-022-01344-w 35706035 PMC9202104

[99] Kumar R, Khandelwal N, Thachamvally R, et al. Role of MAPK/MNK1 signaling in virus replication. Virus Res. 2018;253:48–61. doi: 10.1016/j.virusres.2018.05.028 29864503 PMC7114592

[100] Kumar R, Khandelwal N, Chander Y, et al. MNK1 inhibitor as an antiviral agent suppresses buffalopox virus protein synthesis. Antiviral Res. 2018;160:126–136. doi: 10.1016/j.antiviral.2018.10.022 30393013

[101] Chander Y, Kumar R, Khandelwal N, et al. Role of p38 mitogen-activated protein kinase signalling in virus replication and potential for developing broad spectrum antiviral drugs. Rev Med Virol. 2021;31(5):1–16. doi: 10.1002/rmv.2217 33450133

[102] Chander Y, Kumar R, Verma A, et al. Resistance evolution against host-directed antiviral agents: buffalopox virus switches to use p38-γ under long-term selective pressure of an inhibitor targeting p38-α. Mol Biol Evol. 2022;39(9):39. doi: 10.1093/molbev/msac177 PMC9435063 35975687

[103] Kumar R, Barua S, Tripathi BN, et al. Role of rock signaling in virus replication. Virus Res. 2023;329:199105. doi: 10.1016/j.virusres.2023.199105 36977446 PMC10194102

[104] Kumar N, Sharma S, Kumar R, et al. Evolution of drug resistance against antiviral agents that target cellular factors. Virology. 2024;600:110239. doi: 10.1016/j.virol.2024.110239 39276671

[105] Kumar N, Barua S, Thachamvally R, et al. Systems perspective of morbillivirus replication. J Mol Microbiol Biotechnol. 2016;26(6):389–400. doi: 10.1159/000448842 27607146

[106] Khandelwal N, Chander Y, Rawat KD, et al. Emetine inhibits replication of RNA and DNA viruses without generating drug-resistant virus variants. Antiviral Res. 2017;144:196–204. doi: 10.1016/j.antiviral.2017.06.006 28624461

[107] Kumar N, Khandelwal N, Kumar R, et al. Inhibitor of sarco/endoplasmic reticulum calcium-ATPase impairs multiple steps of paramyxovirus replication. Front Microbiol. 2019;10:209. doi: 10.3389/fmicb.2019.00209 30814986 PMC6381065

[108] Kumar R, Khandelwal N, Chander Y, et al. S-adenosylmethionine-dependent methyltransferase inhibitor DZNep blocks transcription and translation of SARS-CoV-2 genome with a low tendency to select for drug-resistant viral variants. Antiviral Res. 2022;197:105232. doi: 10.1016/j.antiviral.2021.105232 34968527 PMC8714615

[109] Kumar R, Chander Y, Khandelwal N, et al. ROCK1/MLC2 inhibition induces decay of viral mRNA in BPXV infected cells. Sci Rep. 2022;12:17811. doi: 10.1038/s41598-022-21610-9 36280692 PMC9592580

[110] Kumar N, Sharma S, Kumar R, et al. Host-directed antiviral therapy. Clin Microbiol Rev. 2020;33:e00168–19. doi: 10.1128/CMR.00168-19 32404434 PMC7227448

[111] Wang J, Danzy S, Kumar N, et al. Biological roles and functional mechanisms of arenavirus Z protein in viral replication. J Virol. 2012;86:9794–9801. doi: 10.1128/JVI.00385-12 22761375 PMC3446593

[112] Kumar R, Verma A, Kamboj H, et al. H3K27-me3 inhibition induces YTHDF2-mediated decay of m6A-Marked severe acute respiratory syndrome coronavirus 2 transcripts. J Med Virol. 2025;97(4):e70332. doi: 10.1002/jmv.70332 40183255

[113] FAO; UNEP; WHO; WOAH. One health joint plan of action (2022–2026). Working together for the health of humans, animals, plants and the environment. Rome, Italy: FAO ; UNEP ; WHO ; World Organisation for Animal Health (WOAH); 2022. 86. https://openknowledge.fao.org/handle/20.500.14283/cc2289en

[114] Prejit HM, Asha K, Asha K. Effectiveness of One health approach for control of Kyasanur forest disease in Wayanad, Kerala, India. J Vector Borne Dis. 2022;59(1):70–78. doi: 10.4103/0972-9062.331407 35708407

[115] Purse B, Burthe S, Narayanswamy D, et al. A One health ecosystem approach for understanding and mitigating spill-over of tick-borne diseases in India’s degraded forests. One Heal Cases. 2023;2023:1–13. doi: 10.1079/onehealthcases.2023.0011

[116] Mourya DT, Yadav PD, Patil DY, et al. Experiences of Indian council of medical research with tick-borne zoonotic infections: Kyasanur forest disease & crimean-congo haemorrhagic fever in India with one health focus. Indian J Med Res. 2021;153(3):339–347. doi: 10.4103/ijmr.IJMR_532_21 33906997 PMC8204825

[117] Ghosh S, Nagar G. Problem of ticks and tick-borne diseases in India with special emphasis on progress in tick control research: a review. J Vector Borne Dis. 2014;51:259–270. doi: 10.4103/0972-9062.147842 25540956

[118] Nath S, Mandal S, Pal S, et al. Impact and management of acaricide resistance- pertaining to sustainable control of ticks. Int J Livest Res. 2018;8(5):46. doi: 10.5455/ijlr.20180402121612

[119] Eisen L. Personal protection measures to prevent tick bites in the United States: knowledge gaps, challenges, and opportunities. Ticks Tick Borne Dis. 2022;13(4):101944. doi: 10.1016/j.ttbdis.2022.101944 35364518 PMC10859966

[120] Mehla R, Kumar SRP, Yadav P, et al. Recent ancestry of Kyasanur forest disease virus. Emerg Infect Dis. 2009;15(9):1431–1437. doi: 10.3201/eid1509.080759 19788811 PMC2819879

[121] Keshavamurthy R, Charles LE. Predicting Kyasanur forest disease in resource-limited settings using event-based surveillance and transfer learning. Sci Rep. 2023;13. doi: 10.1038/s41598-023-38074-0 PMC10329696 37422454

